# Cribriform pattern in prostate tissues: Predictor for intraductal carcinoma of the prostate based on biopsy and radical prostatectomy pathology

**DOI:** 10.1002/bco2.201

**Published:** 2022-10-20

**Authors:** Ei Shiomi, Renpei Kato, Mitugu Kanehira, Ryo Takata, Jun Sugimura, Yasuyuki Nakamura, Takashi Ujiie, Takaya Abe, Wataru Obara

**Affiliations:** ^1^ Iwate Prefectural Ofunato Hospital Ofunato Japan; ^2^ Iwate Medical University Iwate Japan

**Keywords:** biochemical recurrence, cribriform pattern, Gleason 4, intraductal carcinoma of prostate, predictor

## Abstract

**Objectives:**

This study aims to investigate whether a cribriform pattern on prostate biopsy may be a factor in suspicion of intraductal carcinoma of the prostate after radical prostatectomy.

**Methods:**

This retrospective study assessed 100 men who underwent prostatectomy from 2015 to 2019. Participants were grouped as 76 patients with Gleason pattern 4 and 24 patients without this pattern. All 100 participants underwent retrograde radical prostatectomy and limited lymph node dissection. The same pathologist evaluated all specimens. The cribriform pattern was evaluated with haematoxylin and eosin counterstaining, and intraductal carcinoma of the prostate was evaluated with immunohistochemical analysis of cytokeratin 34βE12.

**Results:**

Patients with intraductal carcinoma of the prostate on immunohistochemical analysis showed a significant tendency to relapse in the postoperative period, and those with the cribriform pattern on biopsy had a significant recurrence rate. In univariate and multivariate analyses, intraductal carcinoma of the prostate confirmed in biopsy tissue was an independent predictor of biochemical recurrence after prostatectomy. The rate of intraductal carcinoma of the prostate confirmation was 28% of cases with a cribriform pattern in biopsy tissue, which was increased to 62% in prostatectomy tissues.

**Conclusion:**

The cribriform pattern in the biopsy tissue may be a predictor for intraductal carcinoma of the prostate.

## 1 INTRODUCTION

The cribriform pattern is an organisational type of Gleason pattern 4 and a strong prognostic marker for distant metastasis and disease‐specific mortality. ^1^, ^2^, ^3^, ^4^, ^5^, ^6^ Intraductal carcinoma of prostate (IDC‐P) is an aggressive form of prostate cancer with histologic and morphologic findings. First proposed in 1985, IDC‐P is considered to be a biologically aggressive form of prostate cancer with prominent architectural and cytologic atypia.^7^, ^8^ This entity is associated with relatively poorer response to surgery and radiotherapy, as well as chemotherapy and androgen deprivation therapy.^7^, ^8^, ^9^, ^10^

Although IDC‐P is evaluated by immunohistochemical analysis,^7^ this approach is time‐consuming, and the understanding of IDC‐P varies among pathologists at each institution.

Many pathologists in other countries are aware of the existence of and even describe IDC‐P. However, the concept itself is not widespread in Japan, and its existence and clinicopathologic significance are only mentioned in prostate cancer guidelines.^11^

Hence, increasing the awareness of IDC‐P in Japan is warranted.

In addition, pathologic assessment is not consistently performed for all cases. A cribriform pattern has been associated with IDC‐P^7^, ^12^, ^13^, ^14^; therefore, this study sought to determine whether IDC‐P in biopsy tissues and radical prostatectomy tissues can be predicted from a cribriform pattern in prostate biopsy tissues.

IDC‐P evaluation may be simplified by prioritising cases with a cribriform pattern instead of performing immunostaining on all cases, if IDC‐P can be predicted from the cribriform pattern.

## 2 MATERIALS AND METHODS

This study sought to examine the relationship between cribriform pattern and IDC‐P in biopsy tissue and prostatectomy tissue. Study participants were 100 men who underwent prostatectomy from 2015 to 2019. Participants were grouped based on Gleason pattern 4: 76 patients had this pattern and 24 did not. All 100 cases underwent retrograde radical prostatectomy and limited lymph node dissection. Postoperative biochemical recurrence was observed in eight cases.

**FIGURE 1:**
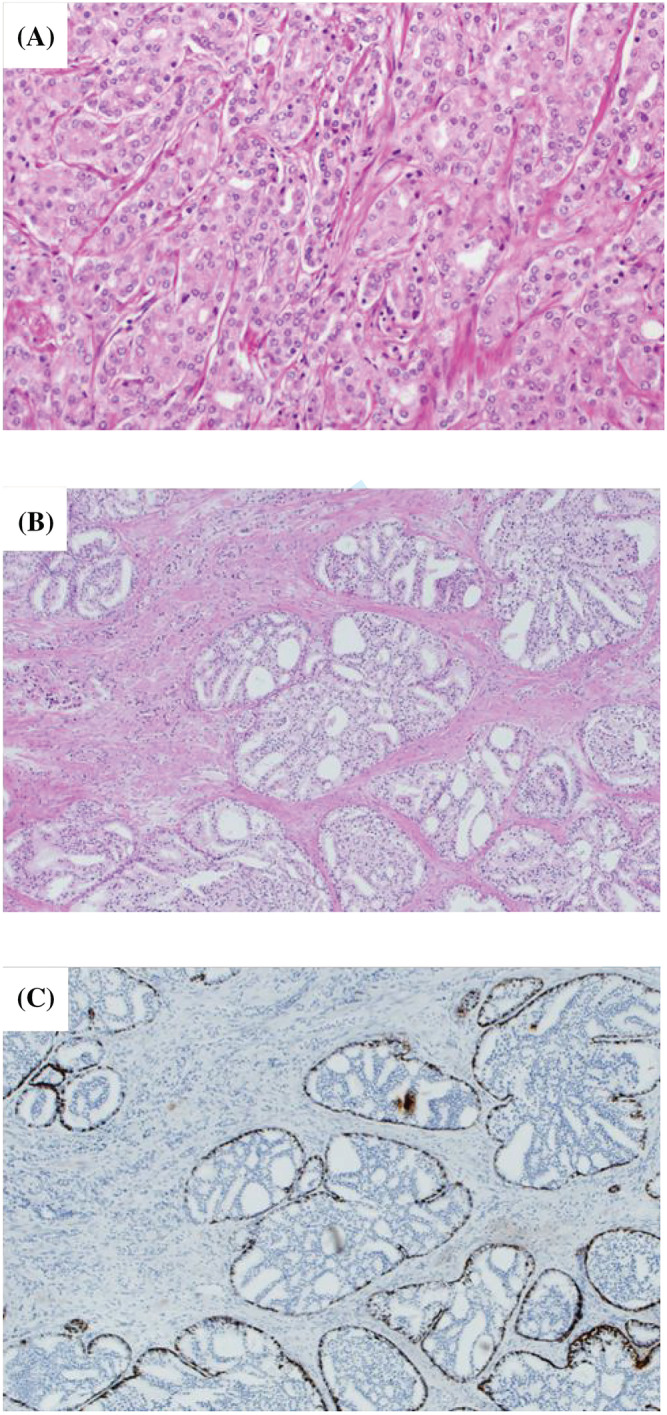
(A) Cribriform pattern with haematoxylin and eosin counterstaining. (B) Intraductal carcinoma with haematoxylin and eosin counterstaining. (C) Intraductal carcinoma with cytokeratin 34βE12

The cribriform pattern was evaluated with haematoxylin and eosin counterstaining, and IDC‐P was evaluated with immunohistochemical analysis of cytokeratin 34βE12 (Figure 1). The definition of biochemical recurrence was prostate‐specific antigen consistently greater than 0.2 ng/ml on more than two assessments over 2–4 weeks.

The same pathologist evaluated all specimens.

Epstein et al. defined IDC‐P as invasive prostate cancer cells invading the lumen of a pre‐existing prostate conduit or adenocarcinoma and growing and spreading on the lumen side.^15^, ^16^ In this study, we have used this definition to evaluate IDC‐P. Immunostaining was added to the sites where cribriform patterns are preferentially present, because cribriform patterns are frequently seen in IDC‐P, and the presence of the basement membrane was checked to determine the presence of IDC‐P.

### 2.1 Statistical analysis

Survival probabilities were estimated using the Kaplan–Meier method. Unadjusted between‐group comparisons for survival time were performed by log‐rank testing. IDC‐P prognostic factor investigation was entered into a logistic regression model to assess the relative contributions. All statistical analyses were conducted with the software package JMP 13 (SAS Institute, Cary, NC, USA). A two‐sided *p*‐value < 0.05 was considered significant.

## 3 RESULTS

Biopsies showed a cribriform pattern plus IDC‐P in 13.2% cases and a cribriform pattern only in 25% cases. Postoperative pathology showed a cribriform pattern plus IDC‐P in 34.2% cases and only a cribriform pattern in 35.5% cases (Tables 1 and 2).

**TABLE 1:** Clinicopathologic characteristics of the cases before prostatectomy and percentage of cribriform pattern and IDC‐P in biopsy tissues

Parameter		Adenocarcinoma, *N* = 76
Median age (range)		73 (59–87)
PSA (%)	<10	58 (76.3)
	10–20	18 (23.7)
	>20	0 (0.0)
Gleason score (%)	3 + 4 = 7	35 (46.1)
	4 + 3 = 7	9 (11.8)
	4 + 4 = 8	30 (39.5)
	4 + 5 = 9	2 (2.6)
cT stage (%)	T1c	32 (42.1)
	T2a	26 (34.2)
	T2b	8 (10.5)
	T2c	7 (9.2)
	T3a	3 (3.9)
NCCN	low	0 (0.0)
	Favourable intermediate	35 (46.1)
	Unfavourable Intermediate	8 (10.5)
	High	33 (43.4)
Cribriform pattern/IDC‐P	+/+	10 (13.2)
	+/−	19 (25.0)
	−/−	47 (61.8)

Abbreviations: cT stage, clinical T stage; IDC‐P, intraductal carcinoma of prostate.

**TABLE 2:** Clinicopathologic characteristics of the cases after prostatectomy and percentage of cribriform pattern and IDC‐P in prostatectomy tissues

Parameter		Adenocarcinoma, *N* = 76
Gleason score (%)	3 + 4 = 7	30 (39.5)
	4 + 3 = 7	11 (14.5)
	4 + 4 = 8	29 (38.6)
	4 + 5 = 9	3 (3.7)
	5 + 4 = 9	3 (3.7)
pT stage (%)	T2a	19 (25.0)
	T2c	41 (54.0)
	T3a	8 (10.5)
	T3b	8 (10.5)
ly (%)	+	2 (2.6)
	−	74 (97.4)
EPE (%)	+	9 (11.8)
	−	67 (64.2)
RM (%)	+	29 (38.2)
	−	47 (61.8)
pn (%)	+	52 (68.4)
	−	24 (17.6)
sv (%)	+	6 (7.9)
	−	70 (67.5)
Cribriform pattern/IDC‐P (%)	+/+	26 (34.2)
	+/−	27 (35.5)
	−/−	23 (30.3)

Abbreviations: EPE, extraprostatic extension; IDC‐P, intraductal carcinoma of prostate; pn, perineural invasion; ly, lymphatic invation; pT stage, pathological T stage; RM, resection margin; sv, seminal vesicle invasion.

**FIGURE 2:**
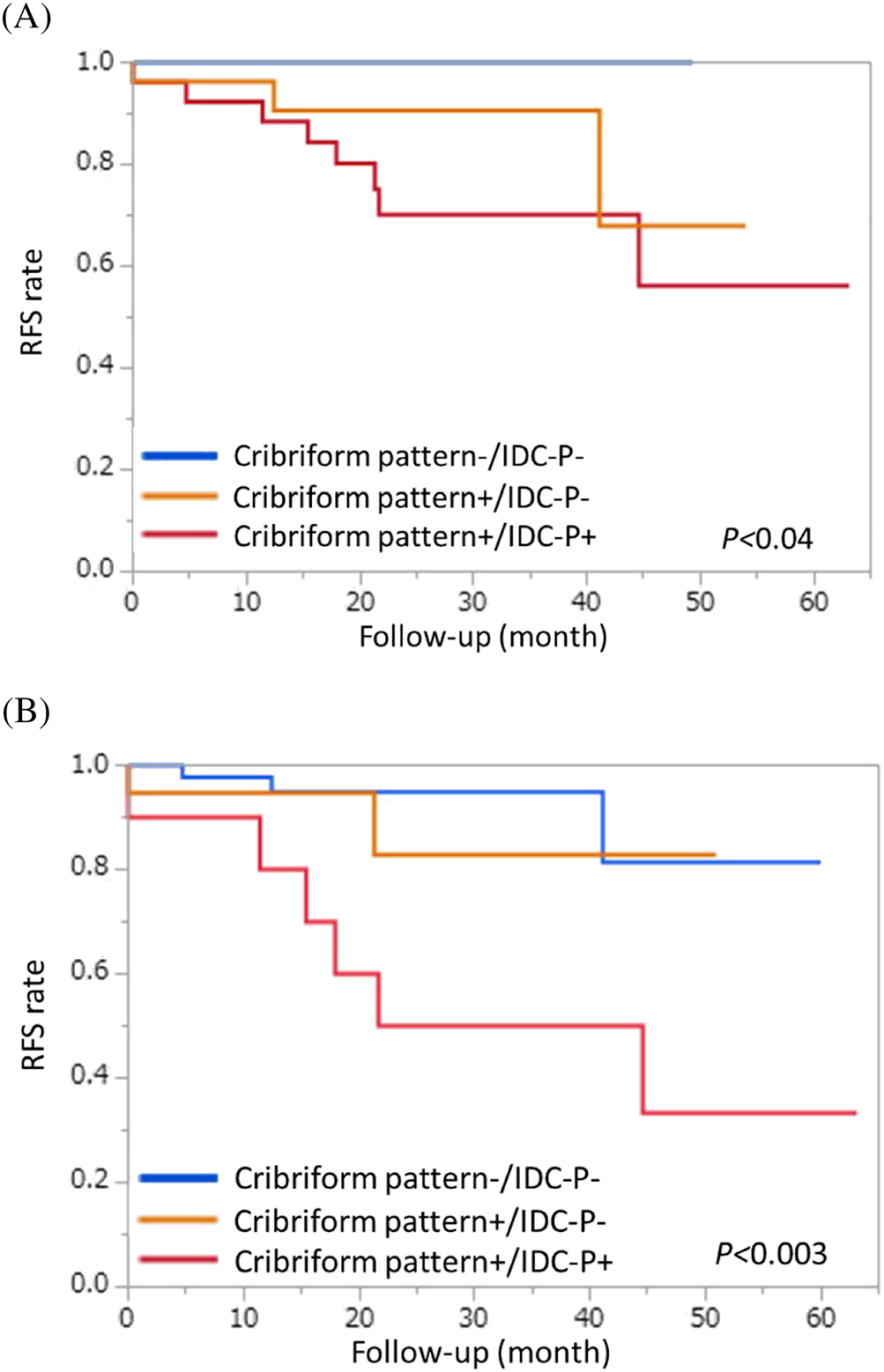
Kaplan–Meier estimates on impact of cribriform pattern and Intraductal carcinoma of prostate (IDC‐P) in recurrence‐free‐survival. (A) After prostatectomy tissues. (B) Biopsy tissues

A comparison of the cribriform pattern and IDC‐P in the prostatectomy tissues showed that the recurrence period tended to be significantly shorter when either the cribriform pattern or IDC‐P was present, as previously reported^7^, ^8^ (Figure 2A). In addition, a biopsy tissue study showed a reduction in the recurrence period only in the presence of IDC‐P (Figure 2B).

Although each risk factor in the NCCN classification is compared with IDC‐P in biopsy tissues, on univariate and multivariate analyses, the detection of IDC‐P in biopsy tissue was an independent predictor of biochemical recurrence of prostate cancer after prostatectomy (Table 3); in comparison with other pathological factors, IDC‐P was most associated with postoperative recurrence (Table 4).

**TABLE 3:** Univariate analysis and multivariate analysis in IDC‐P of biopsy tissues and risk factor of NCCN classification

Risk factor	Univariate analysis		*P*‐value	Multivariate analysis		*P*‐value
	Hazard ratio	95%CI		Hazard ratio	95%CI	
IDC‐P						
+ versus −	10.36	3.118–35.977	0.003	7.535	1.630–38.974	0.0053
PSA						
< 10 versus ≧ 10	0.443	0.130–1.716	0.221	0.467	0.104–0.533	0.347
Gleason score						
< 8 versus ≧ 8	0.191	0.039–0.752	0.0179	0.403	0.078–1.745	0.225
Clinical stage						
< T3 versus ≧ T3	0.435	0.076–8.225	0.4859	0.513	0.088–4.442	0.513

Abbreviations: CI, conflict interval; IDC‐P, intraductal carcinoma of prostate.

**TABLE 4:** Univariate analysis and multivariate analysis in IDC‐P of biopsy tissues and pathological factors

Risk factor	Univariate analysis		*P*‐value	Multivariate analysis		*P*‐value
	Hazard ratio	95%CI		Hazard ratio	95%CI	
IDC‐P						
+ versus −	0.478	0.267–0.855	0.012	0.421	0.209–0.844	0.014
pT stage						
T2 versus T3	0.827	0.414–1.651	0.591	0.255	0.055–1.164	0.078
RM						
+ versus −	1.040	0.668–1.620	0.861	1.203	0.744–1.947	0.449
EPE						
+ versus −	1.488	0.739–2.997	0.266	5.511	1.363–22.274	0.017
SV						
+ versus −	0.779	0.338–1.788	0.554	3.131	0.883–11.095	0.513
pn						
+ versus −	2.169	0.433–10.864	0.340	0.650	0.409–1.031	0.067
ly						
+ versus −	0.893	0.123–6.446	0.910	0.125	0.005–2.964	0.197

Abbreviations: CI, conflict interval; EPE, extraprostatic extension; IDC‐P, intraductal carcinoma of prostate; ly, lymphatic invation; pn, perineural invasion; RM, resection margin; sv, seminal vesicle invasion.

**FIGURE 3:**
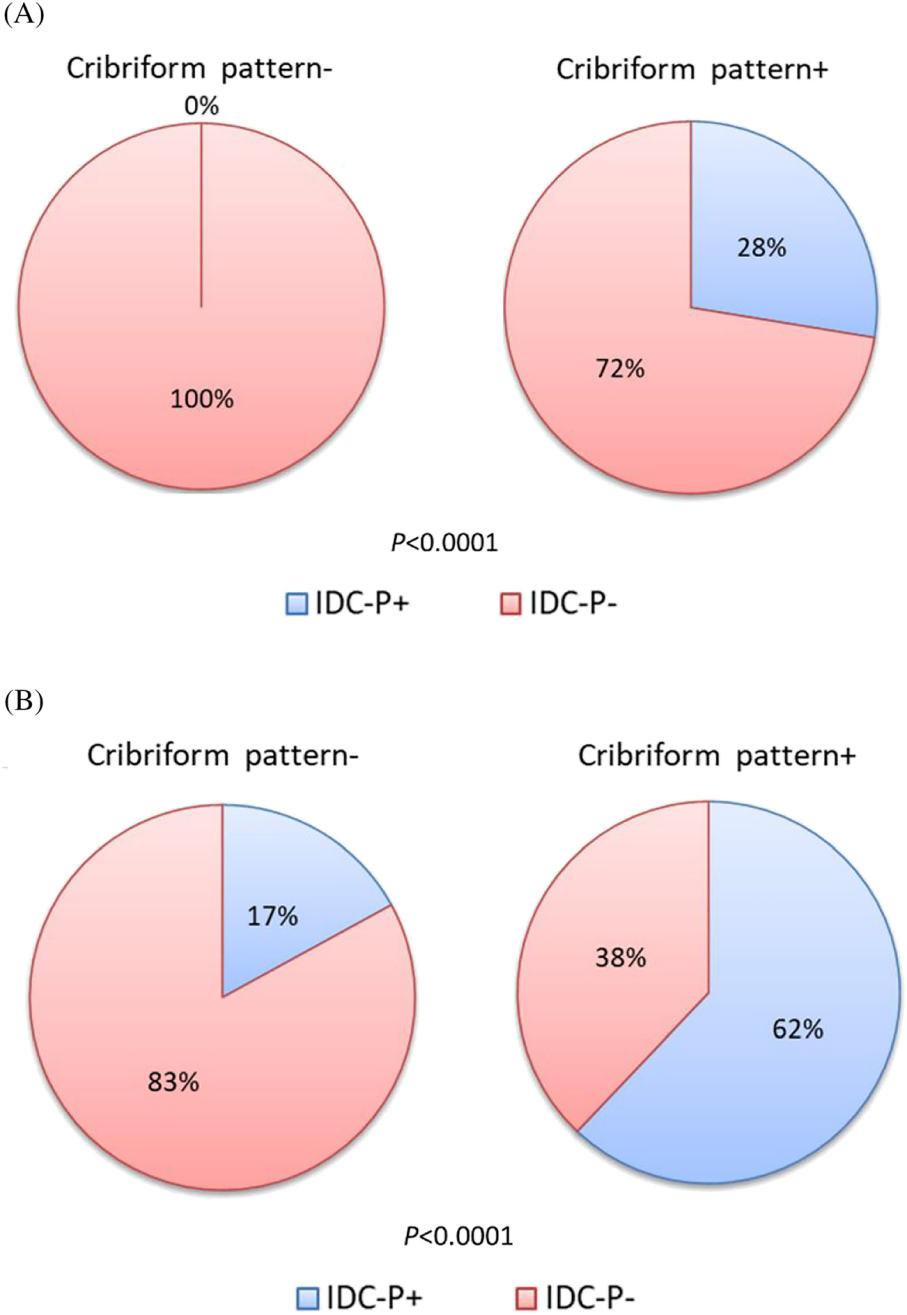
Percentage of Intraductal carcinoma of prostate (IDC‐P) in biopsy tissues and after prostatectomy tissues according to the presence or absence of cribriform pattern in biopsy tissue. (A) Biopsy tissues. (B) After prostatectomy tissues

Of all 76 cases, IDC‐P was confirmed in 28% with a cribriform pattern in biopsy tissue and in 62% with this pattern in prostatectomy tissues (Figure 3).

Additionally, we compared PSA, Gleason Score, clinical stage and the presence of a cribriform pattern in biopsy tissues as preoperative factors predicting IDC‐P in prostatectomy tissues; the cribriform pattern was a predictor of postoperative IDC‐P (Table 5).

**TABLE 5:** Odds ratios for IDC‐P of prostatectomy tissues by cribriform pattern of biopsy tissues, PSA, Gleason score and clinical stage

Risk factor	Hazard ratio	95%CI	*P*‐value
Cribriform pattern			
+ versus −	8.209	2.422–27.818	0.0007
PSA			
< 10 versus ≧ 10	0.271	0.078–0.942	0.0400
Gleason score			
< 8 versus ≧ 8	1.340	0.398–4.513	0.6376
Clinical stage			
< T3 versus ≧ T3	2.877	0.196–42.207	0.4406

Abbreviations: CI, conflict interval; IDC‐P, intraductal carcinoma of prostate.

## 4 DISCUSSION

Results showed that detection of the cribriform pattern and IDC‐P in prostatectomy tissues was related with biochemical recurrence of prostate cancer. Because the presence of IDC‐P was found to be associated with biochemical recurrence in biopsy tissue as well, evaluation for IDC‐P in biopsy tissue may be useful to consider when deciding the treatment policy for this patient population.

The finding of the cribriform pattern in biopsy tissue was not associated with biochemical recurrence. We considered that the reason for the difference in the frequency of detection of the cribriform pattern in biopsy tissue versus prostatectomy tissue was the difference in the amount of tumour collected. However, because the cribriform pattern can be confirmed in about 38% of cases at the time of biopsy—when Gleason pattern 4 can be confirmed as the histologic type in biopsy tissue—it is necessary to actively search for the cribriform pattern as well.

The presence of the cribriform pattern in biopsy tissue was likely to be associated with IDC‐P. In postoperative tissues, the rate of IDC‐P with the cribriform pattern was higher than that without cribriform pattern. It has been reported that the cribriform pattern and IDC‐P are associated with genomic instability and point mutations in TP53, SPOP and FOXA1.^17^ If the cribriform pattern is present, it is highly possible that similar gene mutations are seen in the background, and there is no contradiction in the existence of IDC‐P that is associated with similar gene mutations. Additionally, IDC‐P shows high prevalence of DNA repair gene mutations, including BRCA1/2, ATM and CHEK 2 and among others.^18^ Poly‐ribose‐polymerase (PARP) inhibitors were reported to show response in castration‐resistant prostate cancer, but many of those who do respond were reported to have abnormal DNA repair genes.^19^, ^20^ NCCN guidelines recommend that biopsy tissue with IDC‐P be evaluated for genetic abnormalities, particularly DNA repair genes.

Therefore, IDC‐P evaluation in biopsy tissue is a very important factor in considering future treatment strategies. The possibility of predicting IDC‐P on biopsy and prostatectomy tissues based on the cribriform pattern may ease decision‐making and make it more efficient, such as adding immunostaining for IDC‐P evaluation.

This may be aid at facilities with only one pathologist, such as ours, and it may be one of the reasons to request decontamination when a cribriform pattern is seen in the pathology, from the clinician's point of view. To the best of my knowledge, this is the first study to report IDC‐P prediction using the cribriform pattern.

However, the number of cases in this study is small, and we recommend a more cases and further studies in the future. The cribriform pattern may predict IDC‐P; thus, it may be related to DNA repair genes.

## 5 CONCLUSION

The cribriform pattern may be useful as a predictor for IDC‐P. For patients in which the cribriform pattern is observed in biopsy tissue, evaluation for IDC‐P should be performed both in the biopsy tissue and the postoperative tissue.

## CONFLICT OF INTEREST

We declare that we have no conflicts of interest.

## DISCLOSURE

Approval of the research protocol by an Institutional Reviewer Board and the approval number: R2‐24.

Informed consent: NA.

Registry and the registration no. of the study/trial: NA.

Animal studies: NA.

## AUTHOR CONTRIBUTIONS

Pathology analysis: Yasuyuki Nakamura.
